# Effects of nicotinamide riboside *in ovo* feeding on high-yield broiler performance, meat quality, and myopathy incidence

**DOI:** 10.3389/fphys.2024.1397442

**Published:** 2024-05-20

**Authors:** Clay J. Maynard, John M. Gonzalez, Taketo Haginouchi, Olivia G. Ellis, Ashunti R. Jackson, Casey M. Owens

**Affiliations:** ^1^ Department of Poultry Science, University of Arkansas, Fayetteville, AR, United States; ^2^ Department of Animal and Dairy Science, University of Georgia, Athens, GA, United States; ^3^ Department of Agriculture, Kagoshima University, Kagoshima, Japan; ^4^ Cobb-Vantress Inc., Siloam Springs, AR, United States

**Keywords:** breast myopathies, broiler, meat quality, muscle fiber, vitamin B3

## Abstract

**Introduction:** The objective of this study was to examine the effects of *in ovo* nicotinamide riboside (NR) feeding on high-yield broiler growth and meat quality.

**Methods:** Fertilized Cobb 700 by-product eggs (*N* = 3,240) were randomly assigned to one of four in ovo treatments and injected with 0 (**0NR**), 250 (**250NR**), 500 (**500NR**), or 1,000 (**1,000NR**) mM NR at incubation-day 10. Chicks were hatched, vent sexed, and randomly placed 18 per pen in one of 32 floor pens. On day 48, birds were processed and deboned.

**Results:** There were dose effects for all part weights (*p* < 0.05). Pectoralis major weight of 250, 500, and 1,000NR carcasses were heavier than 0NR (*p* < 0.03) but did not differ from remaining NR doses (*p* > 0.26). Pectoralis minor weight of 250NR carcasses was greater (*p* < 0.01) than 0NR and did not differ from other NR tenders (*p* > 0.21). Pectoralis minor weight of 500 and 1,000NR carcasses was greater than 0NR (*p* < 0.09), but did not differ (*P* = 0.82) from each other. There were no dose effects for all Pectoralis major and minor myopathy scores and incidence except incidence of tenders scoring “0” and “1” for woody-like tender. Percentage of NR1,000 tenders scoring 0 and 1 for woody-like tender were less than and greater than all other treatments, respectively (*p* < 0.05). There were no differences among remaining NR doses and NR0 tenders (*p* > 0.10). There were dose effects for muscle fiber number (*P* = 0.03). There tended to be more muscle fibers within 250 and 1,000NR muscles compared to 0NR (*p* < 0.09). Pectoralis major muscle from 500NR did not differ in muscle fiber number compared to 250 and 1,000NR (*p* > 0.18), but had more (*p* < 0.01) fibers than 0NR muscle. There tended to be more fibers in 250 and 1,000NR muscles compared to 0NR muscle (*p* < 0.09).

**Discussion:** Nicotinamide riboside in ovo feeding caused birds to produce heavier parts; however, myopathy scores and incidence were minimally affected which may have been due greater muscle fiber number.

## 1 Introduction

Over the past 75 years, broiler muscle growth and processing yields have increased substantially. Zuidhof et al. (2014) reported that the broiler industry produced a broiler that utilized half the feed of their ancestors while increasing the breast meat yield by 67%. Since this publication, the industry has advanced these improvements. This is not only economically important but is also very sustainable to continue to meet the ever-growing demand for poultry. Velleman (2019) suggested that genetic selection improvements came from selected birds expressing greater hypertrophic growth. Compared to the ancestral bird, the majority of this growth is due to the modern bird producing more muscle fibers, which are larger (Velleman, 2007). Additionally, although these advancements have improved efficiency and yield, the occurrence of muscle myopathies has become a major issue, which Kuttappan et al. (2016) estimated to cost the industry up to $1 billion per year. Many current myopathy mitigation strategies employed by the commercial industry revolve around managing the bird’s post-hatch growth rate; however, embryonic muscle development and growth manipulation may reduce myopathy incidence and severity.

Embryonic avian muscle development and growth occur in a two-step fashion. Primary myogenesis occurs between embryonic development days 3 and 7 (Ordahl et al., 2001), and secondary myogenesis begins between embryonic development days 8 and 9 (Kahane and Kalcheim, 1998). Secondary fiber formation allows for denser fibers to be present at birth and a greater satellite cell pool (Halevy et al., 2003; Velleman, 2007). Moreover, embryonic satellite cell creation is crucial to supporting post-hatch skeletal muscle growth and repair (Halevy et al., 2000; Halevy et al., 2003). Because the sequence of events associated with myogenesis is routine, many researchers have investigated *in ovo* feeding of numerous compounds to affect muscle fiber and satellite cell formation (Kocamis et al., 1999; Ohta et al., 2001; Grodzik et al., 2013). There has been extensive use of *in ovo* injections to improve the health and performance of poultry. A wide range of substances have been studied, with some showing positive or negative effects, while neutral effects have also been observed (Oliveira, et al., 2023). Growth performance, especially the growth of the *pectoralis major* (breast muscle), has been of great interest. Dayan et al. (2023) used *in ovo* feeding of creatine monohydrate (**CM**) to affect energy reserves and breast muscle development. *In ovo* feeding of CM resulted in a higher breast muscle % at hatching and a higher number of myofibers with a smaller diameter by day 14, suggesting a positive impact on breast muscle development.

The use of nicotinamide riboside (**NR**), a vitamin B_3_ analog, has shown progressive steps in defending against muscle degenerative factors (Mehmel et al., 2020). In the experiment by Zhang et al. (2016), the authors reported that NR-supplemented mice had greater nicotinamide adenine dinucleotide (**NAD** ^**+**^) concentration and satellite cell proliferation. In commercial and high-yield broilers, *in ovo* injection with NR concentrations ranging between 250 and 1,000 nM resulted in increased weight of the hatched-chick *pectoralis major* muscle and muscle fiber density (Gonzalez and Jackson, 2020; Xu et al., 2021; Xu et al., 2022). Xu et al. (2021) also reported that the satellite cell number increased with *in ovo* NR feeding. All the experiments demonstrated NR’s ability to positively influence embryonic muscle development and growth, but its effectiveness on growth performance, meat quality/yield, and myopathy scores were not assessed. Therefore, the objective of the current experiment was to evaluate four concentrations of NR, injected on day 10 of incubation, on broiler performance, carcass characteristics, and muscle myopathy occurrence when grown to 48 days post-hatching.

## 2 Materials and methods

### 2.1 Animal use statement

All animal procedures were approved by the Institutional Animal Care and Use Committee of the University of Arkansas (protocol # 22055).

### 2.2 Egg procurement and incubation

Fertilized Cobb 700 by-product broiler eggs were obtained from a primary breeder (*N* = 3,240; Cobb-Vantress, Siloam Springs, AR; eggs sourced from Blairsville, GA) and transported to the University of Arkansas Poultry Research Farm. Upon arrival, the eggs were kept in a storage cooler at 16°C to be further processed. The eggs were examined, and those possessing cracks, chips, pips, heavy residue, misshapen, or double-yolk eggs were removed. After selection, egg trays with a tray capacity of 84 eggs (Model #PB3179B, Jamesway Incubator Co., Cambridge, Ontario, Canada) were loaded on a trolly and placed in a commercial single-stage incubator (*N* = 3,158; Model #PS500 Incubator; Jamesway Incubator Co., Cambridge, Ontario, Canada) that was set to 37.5°C and 85% relative humidity. On incubation day 10, the trays were removed from the incubator, candled to identify infertile eggs, and then assigned to one of four **NR** injection concentrations in the form of NIAGEN^®^ **(**ChromaDex, Los Angeles, CA, United States).

### 2.3 Injection procedure

Injection procedures were performed according to the protocol of Gonzalez and Jackson (2020). In brief, four concentrations of NR were mixed: 0 (**0-NR**), 250 (**250-NR**), 500 (**500-NR**), and 1,000 (**1,000-NR**) mM NR in a 0.9% saline solution (*n* = 534 eggs per treatment). Eggs were removed from the incubation trays and candled to determine the yolk location. Once the yolk location was determined, 70% methanol was used to clean the exterior surface of the shell’s concave curve, and a 2.54 cm, 20-gauge hypodermic needle was used to pip the shell for injection. The injection needle was inserted approximately 1 cm into the yolk sack, and 100 µL of the solution was slowly depressed to not force amniotic fluid out of the egg. A 1-cm^2^ portion of medical tape (Nexcare, 3M, Maplewood, MN, United States) was then applied over the injection site, and the eggs were returned to the incubator. In an attempt to reduce the thermocycling effects on embryo development upon removal from the incubator, the eggs were held at ∼27°C before injection.

### 2.4 Hatching, sexing, and husbandry

On incubation day 18, the eggs were candled to detect early dead embryos and transferred to hatching baskets (Model #PB600; Jamesway Incubator Co., Cambridge, Ontario, Canada) arranged by treatment and covered with basket tops (Model #PB3179B; Jamesway Incubator Co., Cambridge, Ontario, Canada). A stack of 10 trays was utilized in this design, with an empty basket on the bottom to provide proper airflow according to the manufacturer’s recommendation. The baskets were transitioned to a commercial hatcher (Model #PS500 Hatcher; Jamesway Incubator Co., Cambridge, Ontario, Canada). The conditions were set to meet industry standards, with an average temperature of 36.7°C and a relative humidity of 88%. On day 21, the broiler chicks were removed from the hatcher by treatment and vent-sexed by trained personnel.

Five-hundred seventy-six hatch-day male Cobb 700 by-product broiler chicks were separated by treatment, group-weighed by pen (*N* = 18 birds/pen), and placed into 32-floor pens (1.2 × 1.82 m; ∼0.12 m^2^ per bird; *n* = 8 pens/treatment). The experimental pens consisted of a used litter top dressed with fresh pine shavings, a hanging feeder, and a section of nipple-drinker water line with five nipples. The birds were allowed *ad libitum* access to food and water throughout the trial. Internal research house conditions were held constant at a set point temperature of 32^o^C when placed. The environmental temperature was reduced by ∼2^o^C weekly until an endpoint temperature of ∼17.7^o^C was met. Lighting was maintained from hours of light (**L**) to hours of darkness (**D**) as follows: 24L:0D from day 0–1, 23L:1D from days 1–7, and 16L:8D from days 7–48 (Maynard et al., 2019). A crumbled starter diet was fed from day 0 to day 14, whereas pelleted grower, finisher, and withdrawal diets were fed from 15 to 28, 29 to 42, and 43 to 48 days of age, respectively (Table 1). Bi-weekly body weight (**BW**) and feed consumption (**FI**) were recorded on a pen basis and used to calculate individual body weight gain (**BWG**) and mortality corrected feed conversion ratio (**FCR**). Mortality and the number of culled birds were assessed twice daily.

**TABLE 1:** Experimental starter (0–14 d), grower (15–28 d), finisher (29–42 d), and withdrawal diets (43–48 d) fed to male Cobb 700 by-product broilers from 0 to 48 days of age.

Item, % as-fed	Starter	Grower	Finisher	Withdrawal
Corn	51.64	57.70	60.40	63.71
Soybean meal	41.60	35.43	31.88	29.25
Poultry fat	2.97	3.45	4.56	4.05
DL-methionine	0.40	0.36	0.34	0.31
L-lysine-HCL	0.22	0.26	0.22	0.22
L-threonine	0.16	0.15	0.13	0.12
L-valine	0.04	0.07	0.05	0.04
Dicalcium phosphate	0.89	0.79	0.67	0.64
Limestone	1.25	0.95	0.91	0.87
Salt	0.45	0.45	0.45	0.45
Vitamin and mineral premix^a^	0.15	0.15	0.15	0.15
Choline chloride (60%)	0.09	0.10	0.09	0.10
OptiPhos Plus (1,000 FTU)	0.01	0.01	0.01	0.01
Coccidiostat^b^	0.05	0.05	0.05	0.00
Filler^c^	0.08	0.08	0.08	0.08
Calculated composition, % unless noted otherwise				
AME, kcal/kg	3,000	3,100	3,200	3,200
CP	23.35	21.00	19.50	18.50
Ca	1.00	0.84	0.78	0.75
Available P	0.45	0.42	0.39	0.38
Na	0.18	0.18	0.18	0.18
Digestible Lys	1.32	1.21	1.10	1.04
Digestible TSAA	1.00	0.91	0.86	0.81
Digestible Thr	0.90	0.81	0.74	0.70
Digestible Val	0.99	0.92	0.84	0.79
Digestible Ile	0.87	0.78	0.72	0.68
Digestible Arg	1.44	1.27	1.17	1.10
Digestible Trp	0.26	0.23	0.21	0.20

^a^ The vitamin and mineral premix contained (per kg of complete feed) manganese, 100.0 mg; zinc, 100.0 mg; iron, 50.0 mg; copper, 11.3 mg; iodine, 1.5 mg; selenium, 0.2 mg; vitamin A, 7716 IU; vitamin D_3_, 2756 ICU; vitamin E, 17 IU; vitamin B_12_, 0.01 mg; menadione, 0.83 mg; riboflavin, 6.61 mg; d-pantothenic acid, 6.61 mg; thiamine, 1.10 mg; niacin, 27.56 mg; pyridoxine, 1.38 mg; folic acid, 0.69 mg; biotin, 0.03 mg; and choline, 385.81 mg.

^b^ Supplied 60 g of salinomycin Na per 907.2 kg of complete feed to prevent coccidiosis.

^c^ Sand was considered as filler.

### 2.5 Processing and myopathy scoring

All broilers were individually tagged to maintain their identity throughout processing. Following a 10-h feed withdrawal period, birds from each pen were transported to the University of Arkansas pilot processing plant and individually weighed upon arrival. The birds were hung on inline shackles, electrically stunned (11 V and 11 mA for 11 s), exsanguinated (2 min), scalded in hot water (54.4°C, 2 min), and de-feathered (Mehaffey et al., 2006). Prior to mechanical evisceration, the necks and hocks were removed from each carcass, and the carcasses were weighed to record the hot carcass weight. Carcasses were subjected to a 0.25-h prechill at 12°C before being placed in 0°C immersion chilling tanks for 2.5 hours with manual agitation. At 3-h *postmortem*, the chill tanks were drained, and the carcasses were removed and reweighed to determine the chilled carcass weight. Chilled carcasses were deboned to determine *pectoralis major* (breast) and *pectoralis minor* (tender), wing, whole leg, and rack weights. The part weights were divided by individual back dock live weights to determine the relative part yields. Post-deboning, boneless breast butterflies and tenders were collected for further analysis.

Breast fillets were placed dorsal side down, and muscle thickness, length, and width at one-third the length from the caudal end were measured. Measurements were recorded by a single trained person continuously throughout using a calibrated caliper (Model 500-764-10* IP67, Mitutoyo U.S.A., Aurora, IL) according to the procedures outlined by Maynard et al. (2023).

Boneless breast butterflies were visually scored for white striping (**WS**) and woody breast (**WB**) by a single individual utilizing the palpation method. The measurements ranged from 0 to 3 in increments of 0.5, with 0 being myopathy absence and 3 being the most severe following the scales presented by Kuttappan et al. (2012) and Tijare et al. (2016), respectively. To reduce person-to-person variability, one trained person scored all fillets.

Tenders were scored for woody-like tender (**WT**) and tender feather (**TF**) utilizing the same palpation technique as for breast butterflies. All tenders were evaluated according to Maynard et al. (2023) and classified on a scale of 0–2, with half-point increments. Tenders that expressed no palpable hardness were given a 0 or 0.5 (normal/slight) score, tenders with hardness localized in the cranial region with flexibility in the caudal region received a 1 or 1.5 score, and finally, tenders with complete atrophy from green muscle disease were given a 2 score for WT. A score of 0 was considered if no visible fiber fraying was present or a 0.5 if two splits in the muscle were observed for TF. A score of 1 or 1.5 was considered if moderate fiber fraying was present (>2 split fibers per muscle), and lastly, a score of 2 was considered if severe fiber fraying was present throughout, paired with a soft texture. Attempting to reduce variability, a single trained individual scored all tenders.

Myopathy scoring was collapsed to match a 3-point scale prior to statistical analysis to produce pen occurrences. Scoring ranges were assigned as 0 for scores originally given a 0 or 0.5, 1 for scores originally given a 1 or 1.5, and 2 for scores given a 2, 2.5, or 3 originally.

### 2.6 Meat quality measurements

Butterflies were inverted (dorsal side up) on white plastic storage trays, and the instrumental color was recorded on each left fillet at 24-h *postmortem*. The color was recorded according to American Meat Science Association (AMSA), (2012) guidelines using a Minolta colorimeter equipped with SpectraMagic NX software (Minolta CM-400, Konica Minolta Sensing Americas Inc., Ramsey, N.J., United States). Prior to analysis, the colorimeter was calibrated to CIE specifications using a white calibration tile. Color readings were recorded using the D_65_ illuminant set with a 2-degree observer to decrease sample surface reflectance, and the aperture size was 8 mm. Three separate L*, a*, and b* values were measured for each fillet in the cranial, medial, and caudal locations, which were subsequently averaged.

Following color measurements, pH was calculated in the left-fillet wing-joint portion at 24-h *postmortem* using a calibrated spear-tip pH probe (Model 205, Testo instruments, West Chester, Pennsylvania, United States) inserted into each fillet and allowed to equilibrate for 3 seconds before recording. The pH meter was calibrated to American Meat Science Association (AMSA), (2012) guidelines, utilizing two solutions of 4 and 7.

All breast butterflies were collected, arranged on white plastic storage trays, and wrapped in plastic overwrap film. After 24 h at 4°C, the butterflies were removed, patted dry with a paper towel, and reweighed to determine drip loss. Drip loss was calculated using the percent loss method in relation to deboned-part weight.

Fillets were excised down the center line, individually tagged, reweighed, and placed eight in a vacuum seal bag prior to storage at −20°C in a blast freezer for 21 days. Fillets were removed from the freezer and allowed to thaw for 48 h at 4°C before being patted dry and reweighed to determine relative thawing loss. While weighing, fillets of relative size (within 75 g) were then placed in aluminum foil-covered baking pans, eight to a pan (65 × 395 × 290 mm), on elevated baking racks, and then covered with aluminum foil tented in the center. Fillets were cooked in a commercial convection oven (Model E101-E, Duke Manufacturing Company, St. Louis, Missouri, United States), held at 167°C, and cooked to a final end-point temperature of 74°C, which was verified using a handheld thermometer (Model HT1000 thermometer, Cooper Instruments, Concord, Canada). Fillets were removed from the baking sheet and placed on trays to cool to room temperature (∼21°C) while covered with loose aluminum foil. Fillets were reweighed to determine cook loss as the percent weight from the initial thaw loss weight. After being weighed, fillets were individually wrapped in aluminum sheets (305 × 273 mm) and stored overnight in a 4°C cooler prior to instrumental texture analysis.

Instrumental tenderness was determined using the Meullenet–Owens Razor Shear method presented by Cavitt et al. (2004). Fillets were sheared perpendicular to the muscle fibers in four separate cranial locations using a texture analyzer (Model TA-XT2 Plus, Texture Technologies, Scarsdale, N.Y., United States). A 5-kg load cell using a razor blade with a height of 24 mm and a width of 8.9 mm was set to a 20 mm penetration depth. The machine crosshead speed was set at 5 mm/s and was triggered by a 5 g contact force. Data points were collected with an acquisition rate of 200 points per second, and the shear data included MORS force (MORSF, N) and MORS energy (MORSE, N.mm) values per sample.

### 2.7 Breast cross-sectional area and muscle fiber morphometrics

Breast fillet cross-sectional area (CSA) was collected for two broilers from each pen. Once deboned and scored, fillets were excised down the keel line and again excised down the center of the left fillet at the thickest point to provide the largest footprint of each fillet. Once excised, the largest area was blotted on a sheet of legal paper (22 × 36 cm), outlined with a fine-tip black marker, and a 2.54-cm line was drawn. Papers were scanned using a Xerox scanner (Model #: WorkCentre 5,845; Xerox Corporation, Norwalk, CT; 1,200 × 1,200 dpi), and images were converted to jpeg files for analysis using ImageJ (National Institutes of Health, Bethesda, MD, United States). Images were calibrated using the 2.54-cm line, and the area of the breast fillet (CSA) was measured as the area within the black marker boundary.

Immunohistochemistry analysis was conducted according to Gonzalez and Jackson (2020). In brief, two cryosections (10 μm) were collected on positively charged slides (Diamond White Glass; Globe Scientific Inc., Paramus, NJ., United States) and incubated in 5% horse serum and 0.2% Triton X-100 in phosphate-buffered saline (PBS) for 30 min. Cryosections were incubated at room temperature for 45 min with a blocking solution containing 1:1,000 wheat germ agglutinin-Alexa Fluor 594 (cat no. W11262; Thermo Fischer Scientific, Waltham, MA, United States), washed three times with PBS for 5 min, and 5 µL of 9:1 glycerol in PBS were placed on each cryosection and coverslip for imaging.

Cryosections were visualized at 100-fold magnification using an ECHO Revolve Microscope (ECHO, San Diego, CA, United States). Photomicrographs were collected, stored, and analyzed using ECHO Pro software (ECHO) operating on an iPad Pro (Apple, Cupertino, CA, United States). The cross-sectional area was determined for 1,000 fibers (minimum) as the area within the wheat germ agglutinin border. Muscle fiber number was estimated by dividing the whole muscle CSA by the average muscle fiber CSA (Thayer et al., 2019).

### 2.8 Statistical analysis

Data were analyzed as a completely randomized design, with the pen as the experimental unit for live parameters and myopathy scores, while the bird served as the experimental unit for all other measures. The nicotinamide riboside dose served as the fixed effect, and the pen-analyzed data utilized the pen as the random effect. Data were analyzed with the mixed procedure of SAS 9.4 (SAS Inst. Inc., Cary, NC). Pairwise comparisons between the least squares means of the factor level comparisons were computed using the PDIFF option of the LSMEANS statement. Statistical significance was considered at *p* ≤ 0.05. Tendencies were declared at 0.05 < *p* ≤ 0.10.

## 3 Results

All live performance results for the rearing period until day 47 can be found in Table 2. There were no dose effects for all 0-to-14-day performance characteristics and the remainder of the periods’ FI and FCR (*p* > 0.11). There tended to be dose effects for 0- to 28-day BW and BWG (*p* = 0.09). Broilers from the 250-NR treatment group had greater BW and BWG compared to 0-NR and 500-NR broilers (*p* < 0.04) but did not differ (*p* = 0.24) from 1,000-NR broilers. Body weight and BWG did not differ among 0-NR, 500-NR, and 1,000-NR broilers (*p* > 0.23). There were dose effects for 0–42 and 0–47-day BW and BWG (*p* < 0.05). During both periods, 250-NR broilers had greater BW and BWG than 0-NR broilers (*p* < 0.05) but did not differ from other NR broilers (*p* > 0.10). Broilers from embryos injected with 0-NR did not differ in BW or BWG compared to broilers that received the other NR treatments (*p* > 0.10).

**TABLE 2:** Live performance of male Cobb 700 by-product broilers injected with one of four nicotinamide riboside (vitamin B_3_ complex) concentrations on incubation day 10 and reared to 47 days of age.

	Nicotinamide riboside^a^, mM					
	0	250	500	1,000	SEM	*p*-value
0 to 14						
Body weight, kg	0.39	0.41	0.40	0.40	0.006	0.11
Body weight gain, kg	0.35	0.37	0.35	0.36	0.006	0.12
Feed intake, kg	0.45	0.46	0.44	0.45	0.008	0.46
Feed conversion rate	1.33	1.32	1.33	1.34	0.029	0.95
0 to 28						
Body weight, kg	1.56^a^	1.61^b^	1.55^a^	1.58^a,b^	0.017	0.09
Body weight gain, kg	1.52^a^	1.57^b^	1.51^a^	1.54^a,b^	0.017	0.09
Feed intake, kg	1.99	2.04	1.99	2.04	0.040	0.64
Feed conversion rate	1.35	1.34	1.36	1.37	0.015	0.44
0 to 42						
Body weight, kg	3.21^a^	3.34^b^	3.22^a,b^	3.27^a,b^	0.036	0.05
Body weight gain, kg	3.17^a^	3.30^b^	3.18^a,b^	3.23^a,b^	0.036	0.05
Feed intake, kg	4.52	4.66	4.52	4.62	0.066	0.31
Feed conversion rate	1.47	1.45	1.47	1.47	0.011	0.36
0 to 47						
Body weight, kg	3.87^a^	4.05^b^	3.92^a,b^	4.02^a,b^	0.046	0.04
Body weight gain, kg	3.83^a^	4.01^b^	3.88^a,b^	3.98^a,b^	0.046	0.04
Feed intake, kg	5.63	5.81	5.69	5.77	0.067	0.26
Feed conversion rate	1.50	1.48	1.51	1.50	0.011	0.27

^a,b^Means without a common superscript within a row are significantly different (*p* ≤ 0.05).

^a^ Nicotinamide riboside mixed in 0.9% saline.

There were dose effects for all carcass weights, part weights, and yield percentages (*p* < 0.03), except breast, tender, wing, and rack yields (*p* > 0.14; Table 3). Birds from all NR treatments had greater live and cold carcass weights than 0-NR birds (*p* < 0.05) but did not differ from each other (*p* > 0.16). Carcasses from 250-NR to 1,000-NR had greater hot carcass weights than 0-NR carcasses (*p* < 0.03), while 500-NR carcasses tended (*p* = 0.07) to be heavier than 0-NR carcasses.

**TABLE 3:** Back dock live weight, carcass, and part yields of male Cobb 700 by-product broilers injected with one of four nicotinamide riboside (vitamin B_3_ complex) concentrations on day 10 of incubation and reared to 48 days of age.

	Nicotinamide riboside^a^, mM					
	0	250	500	1,000	SEM	*p*-value
Carcass weights, g						
Live	3,839^a^	3,999^b^	3,944^b^	3,952^b^	39	0.02
Hot carcass	2,938^a,x^	3,080^b^	3,013^a,y^	3,031^b^	32	<0.01
Cold carcass	3,010^a^	3,161^b^	3,094^b^	3,112^b^	32	<0.01
Part weights, g						
Breast	956^a^	1,013^b^	995^b^	994^b^	13	<0.01
Tender	164^a,x^	171^b^	168^a,b,y^	168^a,b,y^	2	0.03
Wing	291^a,x^	307^b^	305^b^	302^a,b,y^	4	0.03
Whole leg	860^a^	894^b^	862^a^	876^a,b^	10	0.05
Rack	717^a^	749^b^	742^b^	745^b^	8	<0.01
Yield^b^, %						
Hot carcass	76.5^a^	77.1^b,x^	76.4^a^	76.6^a,b,y^	0.18	0.03
Cold carcass	78.4^a^	79.1^b,x^	78.5^a^	78.7^a,b,y^	0.15	<0.01
Breast	24.8	25.3	25.2	25.1	0.17	0.14
Tender	4.3	4.3	4.3	4.3	0.03	0.94
Wing	7.6	7.7	7.7	7.7	0.01	0.70
Whole leg	22.3^a^	22.3^a^	21.9^b^	22.1^a,b^	0.13	0.01
Rack	18.7	18.8	18.8	18.9	0.08	0.22

^a,b,c^Means without a common superscript within a row are different (*p* < 0.05).

^x,y^Means without a common superscript by row tended to be different (0.05 < *p* ≤ 0.10).

^a^ Nicotinamide riboside mixed in 0.9% saline.

^b^ Yields based on back dock live weights taken immediately prior to harvest.

Breast and rack weights were greater for all NR treatments compared to 0-NR (*p* < 0.03), but they did not differ from each other (*p* > 0.26). Tender weights from 250-NR carcasses weighed more (*p* < 0.01) than 0-NR tenders but did not differ from 500- or 1,000-NR tenders (*p* > 0.21). Tender weights from 500- and 1,000-NR carcasses did not differ (*p* = 0.82) but tended to be greater than 0-NR tenders (*p* < 0.09). Wing weights from 250- and 500-NR carcasses did not differ (*p* = 0.68) but were greater than 0-NR wings (*p* < 0.02). Wing weights from 1,000-NR carcasses did not differ from 250- or 500-NR wings (*p* > 0.40) but tended to be greater (*p* < 0.06) than 0-NR wings. Whole-leg weights from 250-NR carcasses were greater than 0- and 250-NR weights (*p* < 0.03), but all other comparisons were not different from each other (*p* > 0.21).

Hot and cold carcass yields from 250-NR carcasses were greater than those of 0- and 500-NR carcasses (*p* < 0.01) and only tended to be greater than 1,000-NR carcasses (*p* < 0.10). Hot and cold carcass yields from 0-, 500-, and 1,000-NR did not differ (*p* > 0.11). Whole-leg yields from 500-NR carcasses did not differ (*p* > 0.13) from 1,000-NR yields but were greater than 0- and 250-NR yields (*p* < 0.01). Whole-leg yields from 0-, 250-, and 1,000-NR did not differ (*p* > 0.15).

There were no dose effects for all *pectoralis major* and *minor* myopathy scores (*p* > 0.19), except for tendencies for WT 0 and 1 scores (*p* < 0.08; Table 4). Percentages of 1,000-NR tenders scoring 0 and 1 for woody-like tender were less than and greater than all other treatments, respectively (*p* < 0.05). There were no differences among the remaining treatment groups (*p* > 0.10).

**TABLE 4:** *Pectoralis major* and *minor* myopathies of male Cobb 700 by-product broilers injected with one of four concentrations of nicotinamide riboside (vitamin B_3_ complex) on day 10 of incubation and reared to 48 days of age.

	Woody breast^a^				White striping^b^			
Treatment^c^ (*n* = 8)	Average	0, %	1, %	2, %	Average	0, %	1, %	2, %
0	1.02	42.19	39.12	17.93	1.27	6.93	61.30	31.77
250	1.17	29.53	49.78	20.69	1.42	1.74	54.69	43.57
500	1.14	30.85	47.56	21.59	1.34	3.88	59.54	36.58
1,000	1.16	29.65	42.94	27.41	1.36	7.39	53.95	38.66
SEM	0.064	4.625	0.501	4.450	0.051	1.920	4.082	4.436
*p*-value	0.22	0.14	0.45	0.51	0.19	0.15	0.52	0.32

	Woody-like tender^d^				Tender feathering^e^			
	Average	0, %	1, %	2, %	Average	0, %	1, %	2, %
0	0.37	91.97^a^	8.03^a^	0.00	0.82	48.99	38.38	12.63
250	0.44	89.21^a^	10.79^a^	0.00	0.81	48.10	40.51	11.39
500	0.41	90.60^a^	9.40^a^	0.00	0.74	51.39	41.71	6.90
1,000	0.41	73.32^b^	25.21^b^	1.47	0.82	45.00	42.64	12.36
SEM	0.030	5.294	5.141	0.735	0.065	3.969	3.598	2.475
*p*-value	0.42	0.06	0.08	0.40	0.77	0.72	0.85	0.34

^a,b^Means without a common superscript within a column are different (*p* < 0.05).

^a^ Breast fillets were considered to have a score of 0, 1, or 2 for woody breasts if the fillet was flexible throughout, stiff in the cranial region, or stiff in the cranial and caudal regions, respectively.

^b^ Breast fillets were considered to have a score of 0, 1, or 2 for white striping if the fillet displayed no visible stripes, stripes less than 1 mm, or stripes larger than 1 mm, respectively.

^c^ Injection of 0.9% saline with 0, 250, 500, or 1,000 mmol of nicotinamide riboside.

^d^ Tenders were considered to have a score of 0, 1, or 2 for woody-like tender if the fillet was flexible throughout, stiff in the cranial region, or stiff in the cranial and caudal regions, respectively.

^e^ Tenders were considered to have a score of 0, 1, or 2 for tender feathering if the tender displayed no visible fiber fraying, moderate fiber fraying throughout, or severe fiber fraying throughout the length of the tender, respectively.

There were no dose effects for all raw and cooked meat measures (*p* > 0.11), except for b* value (*p* = 0.02, Table 5). Breast b* values of 500- and 1,000-NR breasts were greater than those of 0-NR breasts (*p* < 0.05) but did not differ (*p* > 0.10) from each other. The b* value of 250-NR breasts did not differ from that of all other treatments (*p* > 0.10).

**TABLE 5:** Raw and cooked meat quality attributes of male Cobb 700 by-product broilers injected with one of four concentrations of nicotinamide riboside (vitamin B_3_ complex) on day 10 of incubation and reared to 48 days of age.

	Nicotinamide riboside, mM					
Parameter (n = 101)	0	250	500	1,000	SEM	*p*-value
Raw measures						
pH^a^	5.91	5.95	5.91	5.90	0.020	0.33
Drip loss, %^b^	0.69	0.76	0.73	0.80	0.054	0.46
Thaw loss, %^b^	6.77	7.07	6.87	7.53	0.275	0.19
L*^c^	56.87	57.61	57.34	57.58	0.258	0.11
a*^c^	3.41	3.25	3.43	3.38	0.131	0.76
b*^c^	9.56^a^	10.06^a,b^	10.15^b^	10.16^a^	0.164	0.02
Cooked measures						
Cook loss^b^, %	25.38	24.96	24.13	25.14	0.640	0.51
MORSF^d^, N	14.07	13.25	13.63	13.84	0.269	0.13
MORSE^d^, N.mm	196.97	186.41	188.89	192.72	3.902	0.19

^a,b^Means without a common superscript by row were determined to be different (*p* ≤ 0.05).

^a^ pH was collected 24 h *postmortem*.

^b^ Drip loss was calculated as percent loss of fillets by weight from deboned weight. Thaw loss was calculated as percent loss of fillets by weight from drip loss weight. Cook loss was calculated as percent loss of fillets by weight from thaw loss weight.

^c^ All color measurements were recorded on the dorsal side of breast fillets 24 h *postmortem*.

^d^ Meullenet–Owens Razor Shear (MORS). F, force; E, energy.

There were no dose effects for whole breast width and thickness (*p* > 0.41), but there tended to be an effect (*p* = 0.08) for length (Table 6). Breasts from 0- and 250-NR birds did not differ from each other or 1,000-NR breasts (*p* > 0.32) but were longer than 500-NR breasts (*p* < 0.04). Breasts from 500- and 1,000-NR birds did not differ (*p* = 0.14) in length. There were no dose effects (*p* = 0.22) for breast muscle CSA, but there were effects for muscle fiber CSA and number (*p* < 0.05). Muscle fiber CSA from 500- and 1,000-NR *pectoralis major* muscles did not differ from each other or 250-NR muscles (*p* > 0.48) but were larger than 0-NR fibers (*p* < 0.03). Muscle fiber CSA from 250-NR *pectoralis major* muscles tended to be larger (*p* = 0.07) than that of 0-NR fibers. There were more (*p* < 0.01) muscle fibers within 500-NR *pectoralis major* muscles when compared to 0-NR muscles, but the number did not differ from 250- and 1,000-NR muscles (*p* > 0.18). There tended to be more muscle fibers within 250 and 1,000-NR muscles compared to 0-NR muscles (*p* < 0.09).

**TABLE 6:** Effects of nicotinamide riboside (vitamin B_3_ complex) dose on incubation day 10 on day-48 post-hatch Cobb 700 *pectoralis major* whole muscle and muscle fiber morphometrics.

	Nicotinamide riboside, mM					
Parameter (*n* = 24)	0	250	500	1,000	SEM	*p*-value
Whole breast morphometrics, cm						
Length	187.8^a^	186.9^a^	182.3^b^	185.6^a,b^	1.58	0.08
Width	106.0	108.3	104.8	106.8	1.46	0.41
Thickness	38.2	39.4	39.6	38.8	0.81	0.65
Muscle fiber morphometrics						
Breast CSA^a^, cm	75.3	77.1	78.9	74.7	1.65	0.22
Fiber CSA^a^, µm^2^	6,187^a,x^	5,453^a,b,y^	5,160^b^	5,340^b^	297	0.05
Fiber number^3^, # × 10^6^	0.81^a,x^	0.98^a,b,y^	1.07^b^	0.95^a,b,y^	0.102	0.03

^a-b^Means without a common superscript by row were significantly different (*p* ≤ 0.05).

^x,y^Means without a common superscript by row tended to be different (0.05 < *p* ≤ 0.10).

^a^ Cross-sectional area; CSA.

## 4 Discussion

The responses in post-hatching BW and BWG observed in the current experiment are the first reported in the literature because previous studies harvested chicks from NR-injected embryos 24 h post-hatching. In all previous studies, BW among the control and birds from NR-injected embryos did not differ (Alcocer et al., 2021; Xu et al., 2021; Xu et al., 2022). The current data agree with this trend as there was no dose effect for day 0–14 BW and BWG; however, it should be noted that the effect was close to a tendency. The treatment effect on BW was apparent during the 0–28-day period, where 250- and 1,000-NR birds were heavier than 0-NR birds by 50 to 20 g, respectively. During the remaining two periods, 250-NR birds were heavier than 0-NR birds by 130 and 180 g, respectively, due to BWG improvements. Similar to foundational chick data (Alcocer et al., 2021; Xu et al., 2021; Xu et al., 2022), there was no BW or BWG advantage when NR was injected above the 250-mM dose. Although the exact biological mechanisms responsible for post-hatch growth remain unknown, current study data indicated that it may have taken more than 14 d of growth for enough protein to be synthesized for the increased muscle fiber formation of NR birds to result in weight differences.

Bekhit et al. (2019) suggested that vitamin B_3_ (niacin) provided fresh meat color stability as a natural antioxidant. In a pilot study, Gonzalez et al. (2019) reported that dietary NR supplementation improved pork redness and color stability. In the current experiment, all broilers supplemented with NR *in ovo* had an increased yellowness but lacked any difference in redness or lightness compared to the control. Given the nature of vitamin B_3_ as an antioxidant, there is reason to believe the vitamin itself provided some objective color variation, but due to the difference only being half a point, visual differences were probably not detectable by the human eye. In addition, the NR salt that was used in the current experiment to produce stock solutions was highly fluorescent in nature, giving it a severe yellow hue. Pigmentation attributed to this solution may have been a direct cause of increased yellowness in NR-injected broilers (Alcocer et al., 2021). In contrast, the relative lightness and redness of the fillets were unaffected, which could be a result of the antioxidant and color stability properties of the vitamin complex. No differences in pH were observed, and no research is currently available at this time to understand the effects of supplemented vitamins on pH progression in broilers.

Meat tenderness is important for consumer satisfaction. Nicotinamide riboside had no effect on the instrumental tenderness of broilers injected in comparison to the control. Dransfield and Sosnicki (1999) originally suggested stacking larger numbers of fibers in an area would produce an increase in toughness for broilers reared for meat production. Furthering this notion, Ebarb et al. (2016) concluded that larger fibers (when fiber density decreased) produced tougher meat compared to smaller fibers (when fiber density increased). When considering the effects of the current experiment and past work by Xu et al. (2021), the number of muscle fibers increased linearly with NR injection concentration to 500 mmol NR. It is possible that the effect of sarcomere shortening due to early deboning (3-h *postmortem*) masked the effects of increased muscle fiber number.

In the first NR study, Gonzalez and Jackson (2020) reported that commercial-yield broilers injected with 250 mM NR as embryos had 38% heavier breasts than birds not injected with NR as embryos. Subsequently, Xu et al. (2021) found there was no increase in breast weight as the NR dose increased from 250 to 1,000 mM, and the weight increase averaged 35% when compared to controls. Using high-yield broilers, Xu et al. (2022) reported that supplementing 100× less NR during embryogenesis increased breast weight by 33%, and they hypothesized that high-yield broilers may require a smaller NR dose than commercial-yield broilers. When grown to market weight, breast weight increased for all birds from NR-injected embryos by 44.6 g (5%) compared to non-injected birds. Similar to chick studies, injecting NR at greater doses did produce an additional increase in breast weight.

The current study provides valuable insight into the *in ovo* feeding effects on the remaining carcass parts. On average, compared to 0-NR parts, injecting embryos with NR caused increases in tender, wing, and rack weights of 5, 14, and 28 g, respectively. Additionally, injecting embryos with 250 mM NR increased whole-leg weight by 34 g compared to 0-NR whole-leg weight. Increases in part weight would produce more salable product volume and return more profit to the industry; however, part weight increases were not surprising because BW increased with NR administration.

The administration of 250 mM NR increased hot and cold carcass weights by 0.6% and 0.7%, respectively, compared to 0-NR carcasses, while the other two NR doses did differ. This difference in NR responses was mainly driven by the whole-leg increase. Except for whole-leg yield, the remaining part yields were unaffected by NR administration in the current experiment. Additionally, NR did not affect breast morphometric measures, but this could mainly be due to the small number measured, given that this was not the main objective of the study. In the work by Fatemi et al. (2021), *in ovo* injection of vitamin D_3_ provided an increase in carcass yield and breast meat yield, with no effect on other carcass part yields. Although not the same vitamin, a breast yield response was not identified with NR injection. Kidd et al. (2004) reported that increasing amino acid density linearly improved breast meat yield. Typically, when a response is noted in leg yield, the opposite effect is noted in breast yield (Kidd et al., 2004). In the current experiment, amino acid concentration was elevated compared to breeder recommendations (Cobb-Vantress, 2019), which should have induced an increase in breast meat yield rather than leg yield. Even so, the response observed in leg yield is not fully understood.

According to Petracci et al. (2015), myopathies began to appear in the industry in the 1980s, and the two most prevalent myopathies, WS and WB, appeared in the 2010s. Condition severity can vary, and both have been associated with heavier, fast-growing birds, especially from high-yield broiler strains (2.72–4.54 kg BW; Kuttappan et al., 2013; Kuttappan et al., 2016; Mueller et al., 2023). Cruz et al. (2017) reported that 32% and 89% of commercial broilers exhibit WB when raised to 35 and 42 days of age, respectively. Therefore, raising broilers to heavier weights and greater ages to meet consumer demand is not attractive in terms of meat quality unless countermeasures to these conditions are developed. Despite increasing breast weight, almost 44 g in the current study, there were no dose effects on WB or WS scores or distributions. Average tender myopathy scores were unaffected by dose, and only 1,000-NR tenders had a 19% decline in “0” tenders with an 18% increase in “1” tenders compared to 0-NR. Using a four-point (0–3) system, Aguirre et al. (2020) demonstrated that the frequency of breasts scoring 2 or 3 increased dramatically when weights were greater than 1,000 g. Although histology was not conducted on tenders to understand the increased incidence of “1” scores, two interesting trends are seen within the data. First, it is encouraging that a dramatic shift in frequency was not seen with heavier breasts produced by NR birds. Second, the greatest increase in breast weight seen by 250-NR breasts did not result in greater WB or WS scores. Third, when NR was injected at smaller NR doses, tender myopathy “0” and “1” scores did not differ from 0-NR scores. Therefore, these results may indicate that NR injection provided a protective mechanism against rampant myopathy formation. Greene et al. (2020) reported that WB fillets had downregulated NAD^+^ content compared to normal fillets, and Xu et al. (2021) found injecting NR at 1,000 mM increased hatched-chick NAD + content. While this could be a protective mechanism, an increased muscle fiber number could also have served as a protective mechanism.

In examining the studies documenting poultry muscle myopathies’ biological origins, one theme becomes clear: enlarged muscle fiber diameter or CSA could be the mechanism initiating the muscle myopathy onset. Dransfield and Sosnicki (1999) were one of the first to report that fast-growing broilers had 42% larger muscle fibers than slow-growing birds. Daughtry et al. (2017) found that large 8-week-old Cobb 500 broilers had approximately 45% larger muscle fibers than small broilers. Additionally, the authors demonstrated that large broilers had a fiber distribution that shifted to the right compared to small broilers; further indicating that muscle fibers were larger in these birds. Clark and Velleman (2017) reported that muscle fibers were the largest in the antero-dorsal portion of the PM muscle, where WB was the most severe. Macrae et al. (2006) suggested that the muscle fibers of myopathy-affected poultry were reaching functional size constraints, which resulted in poor capillary development and reduced efficiency of oxygen delivery and waste removal. In the current study, breasts from NR-injected birds had 15% smaller muscle fibers, which resulted in them having approximately 233,000 (23%) more muscle fibers. While foundational NR studies could not estimate muscle fiber number, fiber density increases ranged between 34% and 75% based on the dose and broiler strain (Gonzalez and Jackson, 2020; Xu et al., 2021; 2022). Based on these results, it could be postulated that NR exhibits an effect on muscle growth and could potentially be utilized to promote muscle hyperplasia in growing broilers.

## 5 Conclusion

The *in ovo* NR feeding had minor performance effects but was able to produce heavier broilers. Producing heavier bird-produced part weights could add millions of pounds of meat available for sale, depending on market conditions. This could result in billions of dollars of extra revenue. There were no detrimental effects on traditional meat quality measurements, and only tender myopathy incidence was negatively affected. Adding 44 more grams of breast weight without greatly affecting myopathy incidence could be due to NR birds’ increase in muscle fiber number, but other mechanisms also need to be explored.

## Data Availability

The raw data supporting the conclusion of this article will be made available by the authors, without undue reservation.
